# Laboratory-based cellular-level correlative visible-light and X-ray microscopy for 3D evaluation of mouse kidney biopsy

**DOI:** 10.1038/s41598-026-44720-0

**Published:** 2026-04-02

**Authors:** Naoki Kunishima, Raita Hirose, Yoshihiro Takeda, Koichiro Ito, Kengo Furuichi, Kazuhiko Omote

**Affiliations:** 1 X-Ray Research Laboratory, Rigaku Corporation, 3-9-12 Matsubara-Cho, Akishima, Tokyo 196-8666 Japan; 2 New Market Development Office, Rigaku Corporation, 3-9-12 Matsubara-Cho, Akishima, Tokyo 196-8666 Japan; 3 Department of Nephrology, School of Medicine, Kanazawa Medical University, 1-1 Daigaku, Uchinada, Kahoku, Ishikawa 920-0293 Japan

**Keywords:** Computed tomography, Nondestructive, Glomerulus, Mesangial cell, Hypercellularity, Biological techniques, Nephrology, Urology

## Abstract

X-ray microscopy (XRM) is an emerging technology for nondestructive evaluation of bio-specimen. We previously reported that paraffin, which is used for normal histology, acts as a tissue contrast enhancement agent in XRM, thereby enabling cellular-level 3D visualization of unstained biological samples. In this work, a new form of correlative visible-light and X-ray microscopy (CLXM) was introduced, where a kidney biopsy sample was first observed by laboratory-based XRM with paraffin-mediated contrast enhancement, and the same sample was then observed by visible-light microscopy (LM) at the same measurement point, to compare images from these two modalities precisely. This laboratory-based cellular-level CLXM was applied to a kidney biopsy from a 5/6 nephrectomy model mouse to evaluate glomerular hypercellularity lesions. The image comparison identified high-opacity particles in XRM images as cell nuclei in various parts of the nephron including glomeruli and tubules. A complementary approach using both LM and XRM images allowed us to identify/segment three lesions as aggregations of mesangial cells. It was found that one mesangial cell occupied a volume of about 100 μm^3^ in these lesions showing different 3D morphology. Present results suggest the availability of laboratory-based XRM for the practical 3D evaluation of whole kidney biopsies at cellular-level spatial resolution.

**Supplementary Information:**

The online version contains supplementary material available at 10.1038/s41598-026-44720-0.

## Introduction

A kidney biopsy is both reliable and essential for clinical diagnosis of kidney diseases^1^. Conventional pathological analysis of a kidney biopsy at cellular-level spatial resolution adopts destructive two-dimensional (2D) methods based on serial sectioning in visible-light microscopy (LM) and electron microscopy (EM) to evaluate pathological changes in glomerular cells such as mesangial proliferation and nephrosclerosis. A major advantage of LM is the availability of various specific staining techniques that enable unambiguous identification of tissue constituents. EM has excellent special resolution. However, because 2D pathological analysis is laborious and destructive, only a small portion of the biopsy is usually evaluated, indicating that the pathological information obtained is quite incomplete. It has also been reported that destructive serial sectioning can deform the original structure of biological sample^2,3^. Furthermore, images from serial sectioning are not necessarily sufficient to elucidate three-dimensional (3D) progression of the pathological changes, because the spatial resolution in the depth direction of the images is no less than the section thickness of about several microns in the case of LM. Although 3D structures at higher resolutions can be reconstructed from serial EM sections using serial block-face scanning EM (SBF-SEM) with submicron-scale thicknesses^4,5^ or focused ion beam scanning EM (FIB-SEM) with nanometer-scale thickness^6,7^, they are also destructive and are too laborious to reconstruct the whole of a kidney biopsy. Selective plane illumination microscopy (SPIM), also called light sheet microscopy, is a practical and nondestructive 3D method in which optical sectioning with several microns of thickness is achieved by illuminating a bio-specimen from the side direction with a thin sheet of light^8,9^. However, SPIM can visualize only fluorescence labeled or autofluorescent components in a bio-specimen, indicating a loss of structural information from the other major parts. Therefore, to make the diagnosis of kidney diseases completer and more informative, another practical and nondestructive 3D method allowing the visualization of entire biopsies at cellular level resolution is desirable.

Recently, we reported that X-ray microscopy (XRM) with paraffin-mediated contrast enhancement enabled cellular-level nondestructive 3D observation of whole biopsies from mouse kidney, in which formalin-fixed paraffin-embedded (FFPE) biopsies were observed by laboratory-based XRM using Cu-target X-rays^10^. In the paraffin-mediated contrast enhancement, paraffin, which is used for normal histology, acts as a negative contrast agent for unstained bio-specimens. The paraffin-mediated contrast enhancement can be generalized as “wax contrast imaging” for XRM, where wax refers to a lipophilic compound that is solid at room temperature and has a melting point of 40–80 °C^11^. Examples of wax include paraffin, other petroleum waxes, and synthetic waxes. Thus, laboratory-based XRM is a strong candidate for a practical and nondestructive 3D method that visualizes whole biopsies at cellular-level spatial resolution. However, unfortunately, a standalone use of the paraffin-mediated contrast in laboratory-based XRM is currently insufficient for the clinical diagnosis of kidney diseases, because the contrast is derived from small differences in electron density of light elements comprising FFPE biopsies and is still weak to clearly identify some of obscure glomerular cells. Therefore, in this work, we introduce a new practical form of correlative visible-light and X-ray microscopy (CLXM), where laboratory-based XRM images of an FFPE biopsy from mouse kidney at cellular level resolution are compared precisely with corresponding LM images of the same measurement points of the same sample. The complementary use of LM and XRM images successfully allowed segmentation of lesions in a kidney biopsy from a disease-model mouse, which provided their cellular-level 3D morphology and volumetric information for the first time.

## Results

### Quality improvement of XRM imaging

**Fig. 1:**
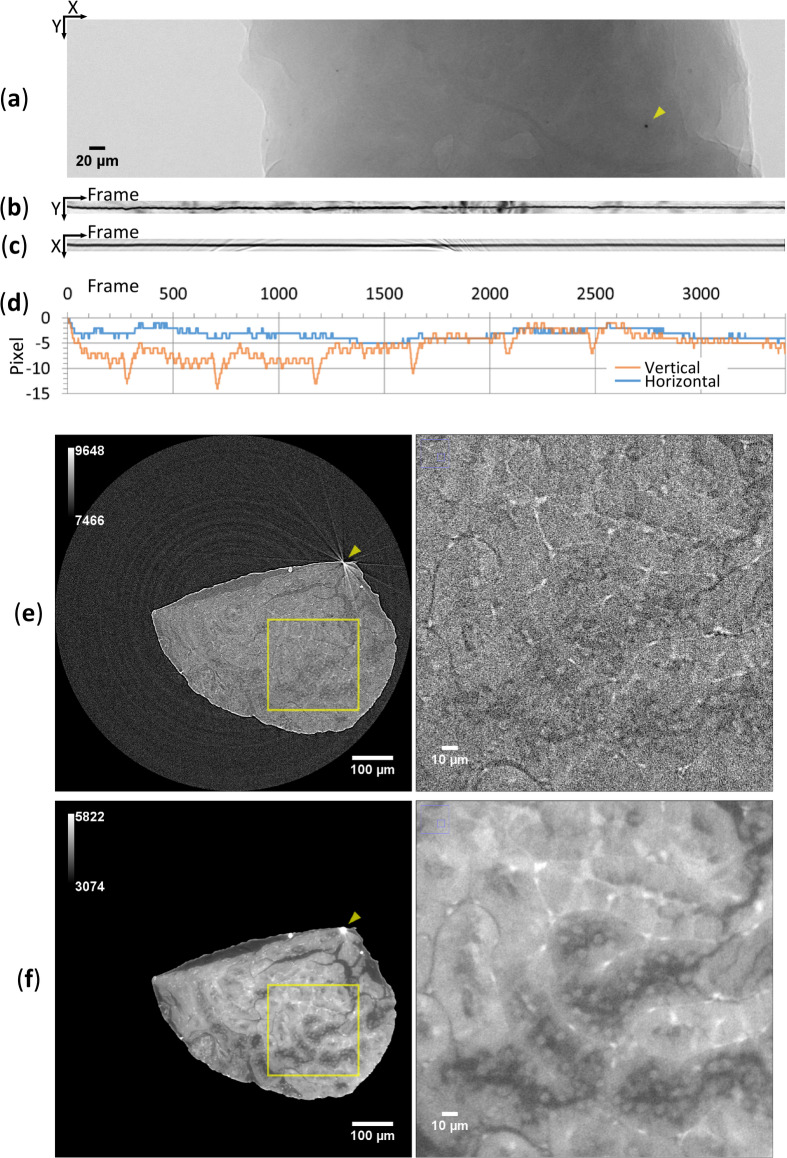
Quality improvement of XRM imaging. (**a**) Projection image of mouse kidney biopsy from disease-model mouse. The positional marker (putative diamond particle) is indicated by an arrowhead. (**b**) Vertical marker trajectory. Black line should be straight if there are no vertical movement of marker during data acquisition. (**c**) Residual horizontal marker trajectory. Black line should be straight if there are no horizontal movement of marker during data acquisition. (**d**) Compensation values for positional shifts. Compensation values to cancel the vertical and horizontal shifts are plotted in pixel against the frame number. (**e**,** f**) Corresponding CT slices after reconstruction without (**e**; 506/726) and with (**f**; 500/709) drift-correction/phase-retrieval. Entire (left; with gray-level scale bars) and partial enlargement of regions indicated by yellow squares (right) are shown. A putative diamond microparticle used for the drift correction is indicated by an arrowhead. Radial streak noises due to sample movement are seen around the marker in (**e**). All figures except for (**d**) were produced using ImageJ.

**Fig. 2:**
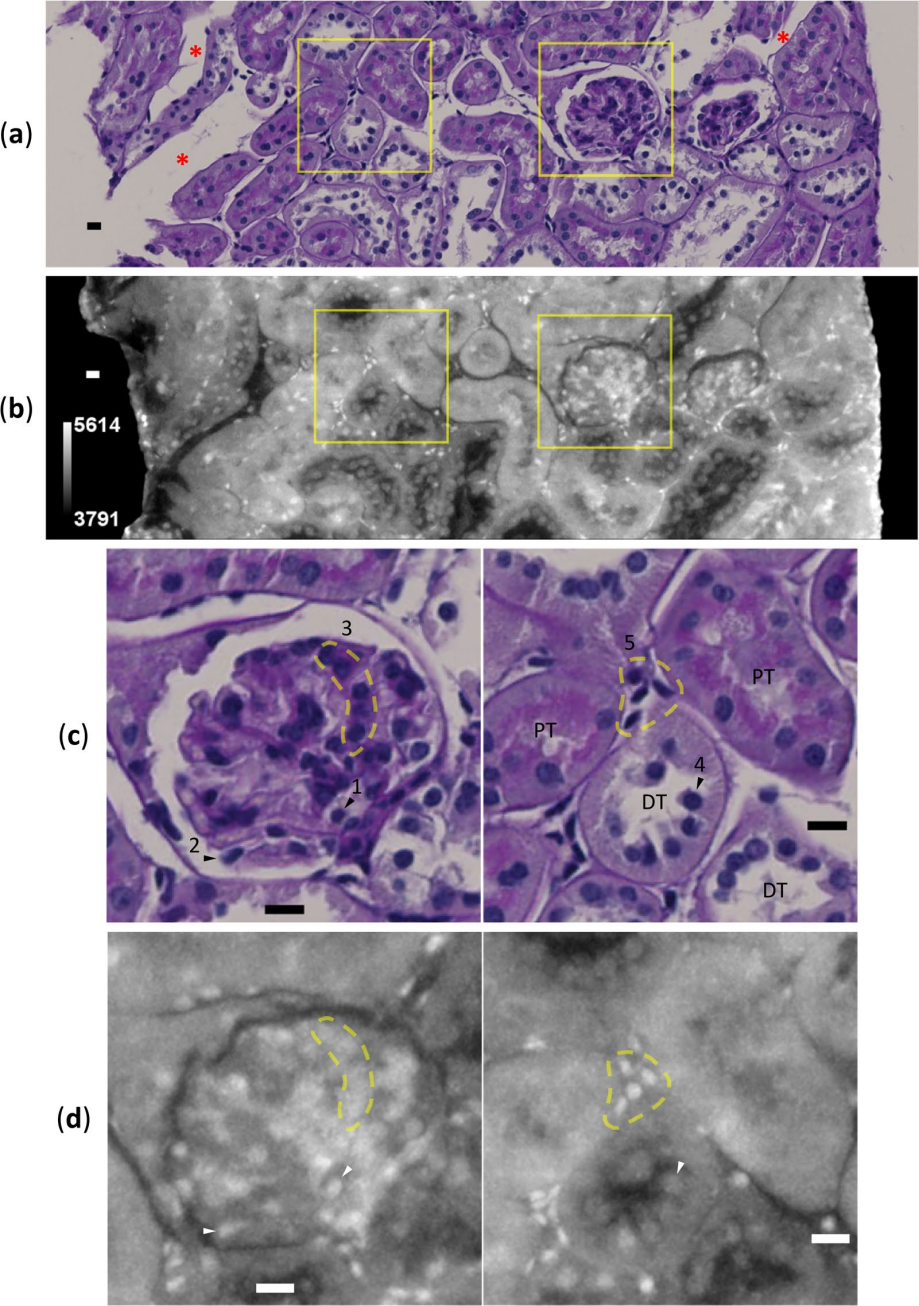
CLXM observation of kidney biopsy from disease-model mouse. Scale bars: 10 μm. Corresponding images of (**a**) LM and (**b**) XRM with gray-level scale bar are shown. The viewpoint of the XRM image was precisely matched to that of the LM image. To compare with 4 μm-thickness of the LM image, the aligned XRM image was integrated by 15 slices using “Grouped z-projection” tool (maximum value) in ImageJ. Of 132 slices produced, the 39^th^ slice was compared. Obvious local discrepancies between the two images are indicated by three red asterisks on the LM image. (**c**, **d**) Magnified corresponding parts indicated by yellow squares in (**a**, **b**) around the glomerulus (left) and tubules (right). Abbreviations PT and DT denote proximal and distal tubules, respectively. Corresponding tissue constituents are labeled: 1 for a nucleus of putative vascular endothelial cell; 2 for a nucleus of glomerular podocyte; 3 for a normal glomerular mesangial region comprising mesangial cells and mesangial matrices; 4 for a nucleus of tubular epithelial cell in DT; 5 for nuclei of putative renocortical interstitial cells. All figures were produced using ImageJ.

For the cellular-level comparison between LM and XRM images of kidney biopsy, the quality of XRM imaging had to be improved. One of main reasons for this deterioration of XRM image is rigid-body movement of the sample due to temperature fluctuation during data acquisition, which can be improved by a drift correction of projection data after the X-ray scanning. In this work, the drift correction was performed using a putative diamond particle as a positional marker (Fig. 1a), where the coordinates of projection images were translated to cancel the marker movement. The drift correction was performed on a case-by-case basis because the pattern of the rigid-body movement was difficult to be controlled. In the case of the disease-model mouse, the widths of the marker movement in the vertical and horizontal directions were evaluated as 3.7 μm and 1.3 μm, respectively (Fig. 1b–d). Thus, only the major vertical shifts were considered in the drift correction, which effectively reduced streak noises around objects in the XRM images (arrowheads in Fig. 1e,f). On the other hand, in the case of the normal mouse, the widths of the marker movement in the vertical and horizontal directions were evaluated as 2.8 μm and 2.6 μm, respectively, and both vertical and horizontal shifts were considered in the drift correction. These drift-corrected projection data were further treated by propagation-based phase retrieval, which improved the contrast-to-noise ratio (CNR) between tissue and paraffin by over 10 times (Table 1). As a result, the drift correction and the phase retrieval provided high-quality XRM images allowing observation of cell nuclei and glomerular mesangial regions in the kidney biopsy (Figs. 1e,f, 2d).

**Table 1:** Observation statistics.

Type	Treatment	Transmission^a^	SBE (μm)^b^	CNR^c^	
				Air-Paraffin	Paraffin-Tissue
Disease model	Original	0.847	NA^d^	1.14 ± 0.13	0.63 ± 0.13
	Drift-corrected /phase-retrieved		0.58 ± 0.07	27.9 ± 3.7 *P* = 1.5 × 10^−4^	11.1 ± 1.3 *P* = 7.1 × 10^−5^
Normal	Original	0.881	NA^d^	1.39 ± 0.17	1.46 ± 0.54
	Drift-corrected /phase-retrieved		0.55 ± 0.07	45.1 ± 2.1 *P* = 2.1 × 10^−6^	24.1 ± 1.7 *P* = 2.7 × 10^−6^

A value with range denotes an average with a 95% confidence interval from five independent measurements. ^*a*^Calculated value from the intensity histogram of the entire raw projection data; the peak intensity of tissue was divided by that of air. ^*b*^Size of blurring at edges^10^ of the paraffin-sample boundary from 6–10 μm line opacity profiles in a CT slice (361/709) for the disease-model mouse and from 5–6 μm line opacity profiles in a CT slice (1112/2168) for the normal mouse. ^*c*^Contrast-to-noise ratio^10^ calculated from an opacity comparison between two selected regions (15 pixels × 15 pixels) in the same CT slice; indicated materials were compared. Average CNR values of original and drift-corrected/phase-retrieved CT regions were compared by *t*-test for each result (*P*-values), according to the procedure described in Methods. ^*d*^Not available due to high level of noise in opacity profiles.

### Alignment/comparison of LM and XRM images

High-density particles in XRM images were clearly identified as cell nuclei, because the positions of the cell nuclei in LM images and those of the high-density particles in XRM images coincided in many cases. To enable a cellular-level image comparison, XRM images were aligned precisely with LM images using the cell nuclei as positional markers (Fig. 2a,b). The accuracy of this alignment procedure depended on the size of CT slices, with the average residuals in orientation angle being within 1° (Supplementary methods). As a result, many more nuclei common to both LM and XRM images were identified in various nephron parts: epithelial cells in the distal tubule, constituent cells in the glomerulus including podocytes and putative glomerular endothelial cells, leucocytes such as lymphocytes in vessels, and putative renocortical interstitial cells (Fig. 2c,d). In these nephron parts, cell nuclei are clearly observable, indicating low background density in the regions. Nuclei of glomerular mesangial cells are also observable but only moderately, because they are surrounded by mesangial matrices with moderate density. On the other hand, cell nuclei in the proximal tubule are not clearly observed, probably due to the high background density of surrounding heavy materials such as mitochondria. The opacity of cell nuclei in XRM images may also depend on the elemental composition of the nuclei themselves. For instance, since it is known that the DNA content of a cell nucleus changes as the cell cycle progresses^12^, fluctuations in the content of DNA, a heavy substance, are likely to affect the opacity of cell nuclei in XRM images. In addition, the LM images showed widespread local distortion of kidney tissue compared to the corresponding XRM images. This distortion may have been caused by mechanical perturbations during LM sample preparation (Fig. 2a,b). However, whether the structures seen in the XRM images are more physiologically relevant than those from LM is not conclusive and should be clarified in the future.

### Segmentation and volume rendering of glomerulus

**Fig. 3:**
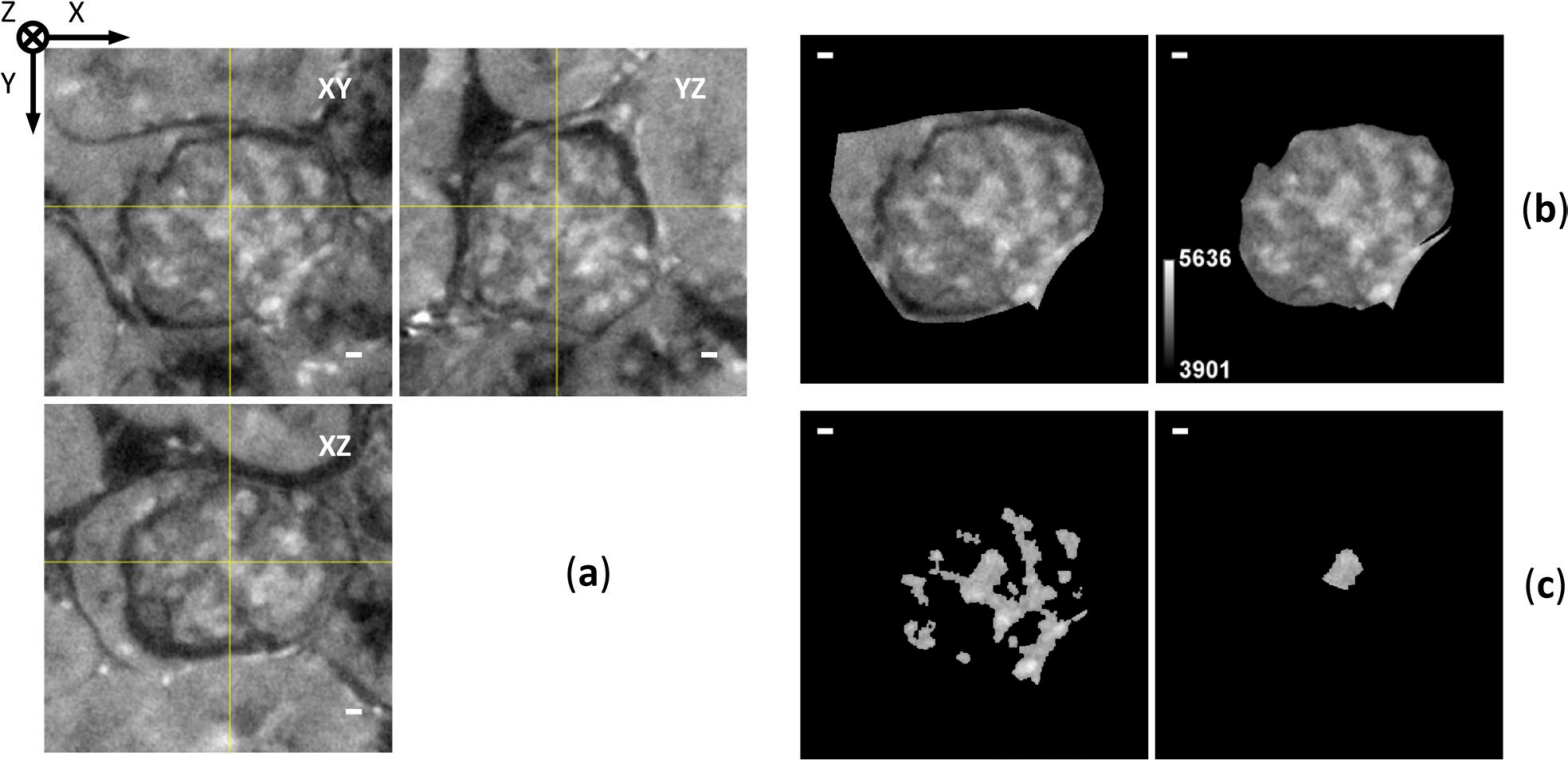
Segmentation of glomerulus from disease-model mouse. Scale bars: 5 μm. (**a**) Three orthogonal CT slices by cutting original XRM image along yellow lines indicated. The XY slice corresponds to slice 182/400 of the original XRM image. The perspective of the XY slice is like that in Fig. 2. Glomerular lesion 1 is shown in the center of all three slices. Segmentation procedures on the XY slice are shown for (**b**) glomerulus with (left) and without (right; with gray-level scale bar) Bowman’s capsule, and for (**c**) all (left) and lesion 1 part (right) of high-density regions in glomerulus without Bowman’s capsule. The segmentation was performed manually except that the high-density regions were segmented by binarization. All figures were produced using ImageJ.

**Fig. 4:**
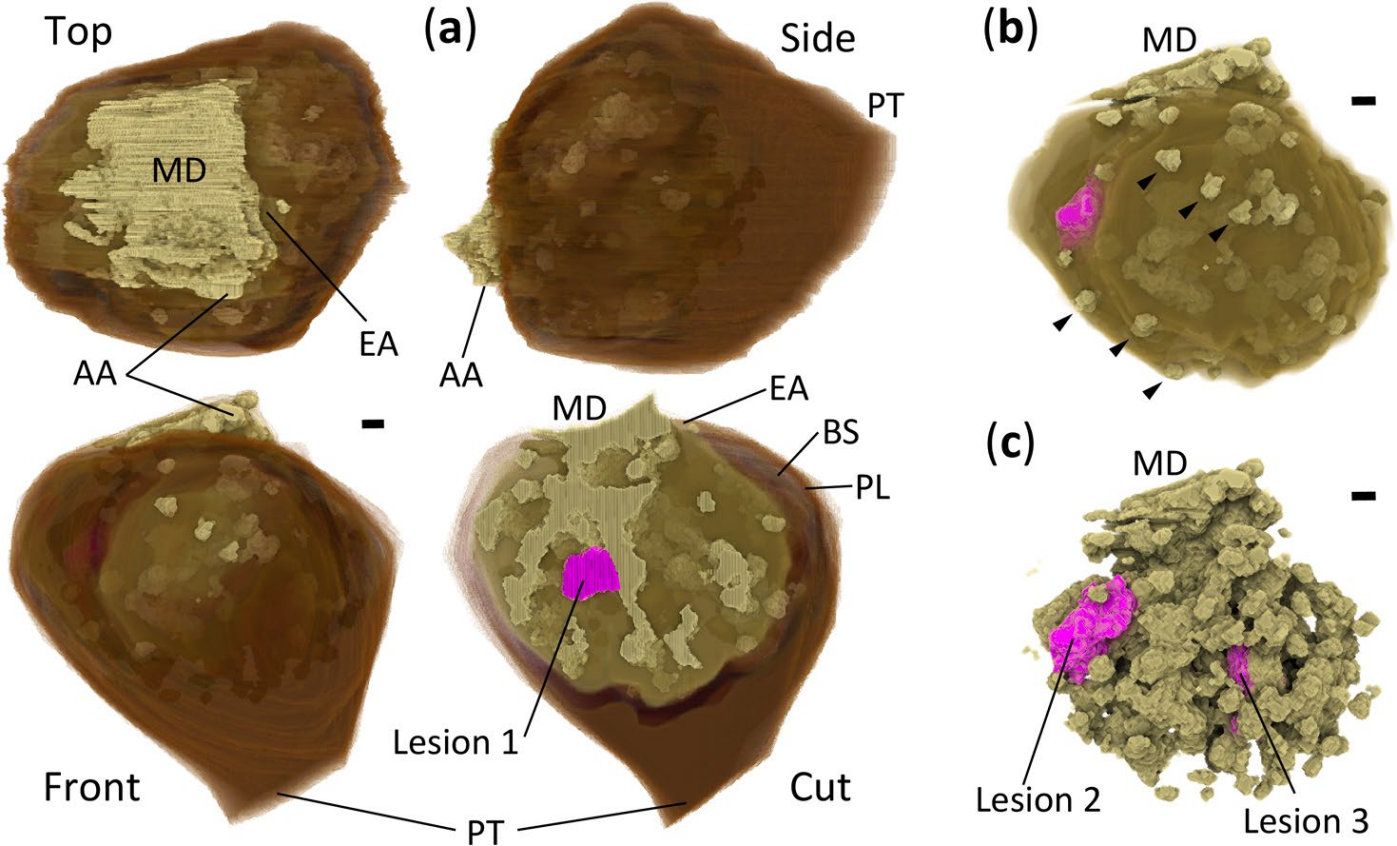
Volume rendering of glomerulus from disease-model mouse. Scale bars: 5 μm. (**a**) Volume rendering of the glomerulus with Bowman’s capsule. Presented are three models from orthogonal perspectives (front, top, side) and a cut model from similar perspective as that of the front model. The perspective of the front model is the same as that of the CT slices in Fig. 3b,c except for an in-plane rotation. Important parts are labeled: Bowman’s space (BS), parietal layer (PL), afferent arteriole (AA), efferent arteriole (EA), macula densa (MD), proximal tubule (PT). The colour scale from brown to light brown denotes low to high density (X-ray opacity) in the regions, respectively. Parallel lines on the model surface are artifacts due to a technical limitation of the manual segmentation. The cut model is cut by the same plane as that of the CT slices in Fig. 3b,c so that the same structures inside the glomerulus are seen. Opaque and translucent regions inside the glomerulus represent high-density and the other regions, respectively. Glomerular lesions are coloured magenta. Additional volume renderings of the glomerulus without Bowman’s capsule: (**b**) whole and (**c**) high-density regions. The perspective is the same as that of the front model in (**a**). Labeling and colouring are the same as those used for the cut model in (**a**). Some of the podocyte nuclei found on the glomerular surface are indicated by arrowheads. All figures were produced using Drishti.

**Fig. 5:**
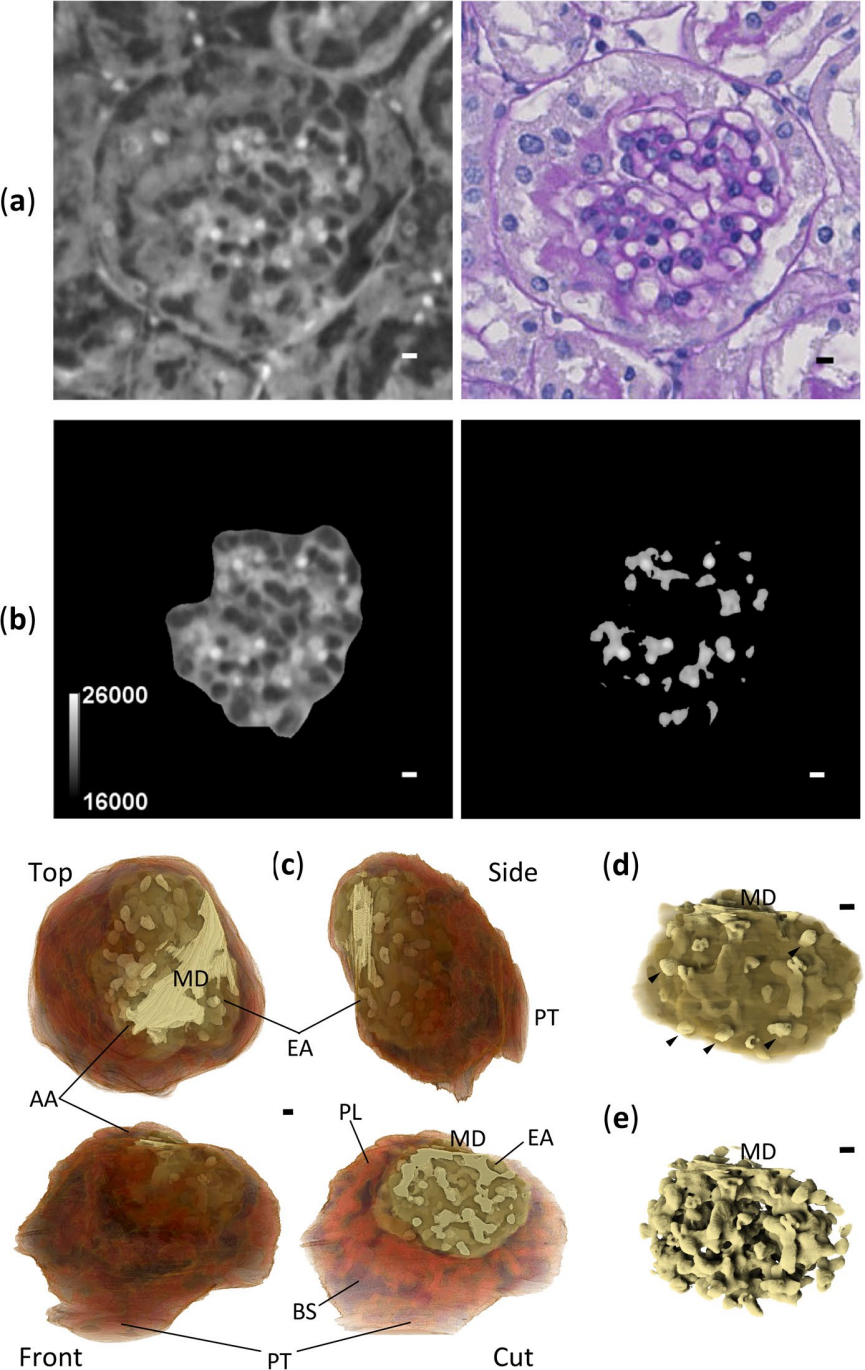
CLXM observation of kidney biopsy from normal mouse. Scale bars: 5 μm. (**a**) CT slice 415/703 of the aligned XRM image (left) with corresponding LM image (right). Image integration by 15 CT slices was not applied to the XRM image. (**b**) High-density regions (right) of the segmented region of the glomerulus without Bowman’s capsule (left; with gray-level scale bar) on CT slice 415/703. (**c**) Volume rendering of the glomerulus with Bowman’s capsule. Presented are three models from orthogonal perspectives (front, top, side) and a cut model from the same perspective as that of the front model. The perspectives and colouring are comparable to those in Fig. 4a. Important parts are labeled in the same manner as those in Fig. 4a. Additional volume renderings of the glomerulus without Bowman’s capsule: (**d**) whole and (**e**) high-density regions. The perspective is the same as that of the front model in (**c**). Labeling and colouring are the same as those used for the cut model in (**c**). Some of the podocyte nuclei found on the glomerular surface are indicated by arrowheads. Figures (**a**, **b**) and (**c**–**e**) were produced using ImageJ and Drishti, respectively.

To investigate 3D structure of glomerulus from a disease-model mouse, segmentation and volume rendering of XRM images were performed. For the segmentation of glomerulus (Fig. 3a,b, Supplementary Videos S1–S3), PAS-stained basement membranes in LM images were used as a positional guide (Fig. 2c). In XRM images, mesangial regions comprising mesangial cells and mesangial matrices showed higher X-ray opacities, indicating their higher densities compared to other parts (Fig. 2d). In some glomeruli, lesions showing “hypercellularity,” an aggregation of mesangial cells, were observed in both LM and XRM images. Glomerular hypercellularity is a well-known lesion in the 5/6 nephrectomy model^13^. To evaluate the utility of XRM in the diagnosis of kidney diseases, we examined whether the hypercellularity lesions could be identified/segmented in 3D as parts of glomerular high-density regions in XRM images (Fig. 3a,c, Supplementary Videos S4–S7). Because the nuclei of mesangial cells were only marginally observable in XRM images, information from corresponding LM images was essential for the correct identification/segmentation of lesions. To investigate the 3D progression of pathological changes, a volume rendering of the glomerulus with the segmented lesions was drawn (Fig. 4, Supplementary Video S8). The high-density region exhibits a tree-like structure with many branches beginning at the macula densa. The lesions look like protrusions developed from the normal tree-like mesangial regions (lesions 1–3 in Fig. 4). Such protrusions were not observed in glomeruli from a normal mouse (Fig. 5c–e, Supplementary Videos S9–S13). Instead, a more extended vascular network and a larger parietal layer with reticulated Bowman’s space were observed (Fig. 5, Table 2). Unfortunately, because this work is based on the analysis of a single glomerulus from a representative mouse, statistical evaluation of the results is not available. In addition, Table 2 shows differences in opacity between normal and disease-model mice, which is likely due to a sensitivity difference of detectors used for the data collection. The normal mouse data collected with CMOS detector showed slightly better image quality when compared to the disease-model mouse data collected with CCD detector (SBE and CNR in Table 1). However, since no cell aggregation (i.e. lesion) was detected for the normal mouse observation even when using the high-quality CMOS detector, it is likely that similar results would be obtained by replacing the detector with CCD.

**Table 2:** Volumetric analysis of glomeruli from mouse kidney.

Type	Regions^a^	Volume (μm^3^)^b^	Ratio versus			Opacity	
			GB	GM	HDR	Mean^c^	Range
Disease model	GB	208,000	1	–	–	4700 (244)	4000–5830
	GM	120,000	0.574	1	–	4770 (222)	4080–5830
	PL	55,600	0.267	–	–	4740 (183)	3960–5650
	BS	33,100	0.159	–	–	4380 (136)	3900–5190
	HDR	35,000	–	0.293	1	5050 (123)	4900–5830
	Lesion 1	486	–	–	0.014	5030 (95)	4830–5380
	Lesion 2	825	–	–	0.024	5070 (124)	4830–5580
	Lesion 3	497	–	–	0.014	5040 (92)	4830–5470
Normal	GB	480,000	1	–	–	20,300 (1288)	16,500–27,200
	GM	141,000	0.293	1	–	20,600 (1500)	17,000–27,200
	PL	224,000	0.466	–	–	20,800 (794)	19,600–27,200
	BS	116,000	0.241	–	–	18,900 (489)	16,500–19,700
	HDR	32,800	–	0.233	–	22,700 (821)	21,700–27,200

^a^Abbreviations used are: glomerulus with Bowman’s capsule (GB), glomerulus (GM), parietal layer (PL), Bowman’s space (BS), high-density regions in glomerulus (HDR). HDR are subregions in GM with opacities higher than 4900 for the disease-model mouse and 21,750 for the normal mouse, corresponding to 46.9% level of the GM opacity range. We searched for the threshold conditions and found that 46.9% of the relative threshold could extract the most continuous and the least overlapping mesangial regions. ^*b*^Multiplied value of the voxel volume (0.0199 μm^3^ for the disease-model mouse and 0.00635 μm^3^ for the normal mouse) by the number of voxels. ^*c*^Population standard deviation is given in parenthesis.

### 3D analysis of glomerular lesions

**Fig. 6:**
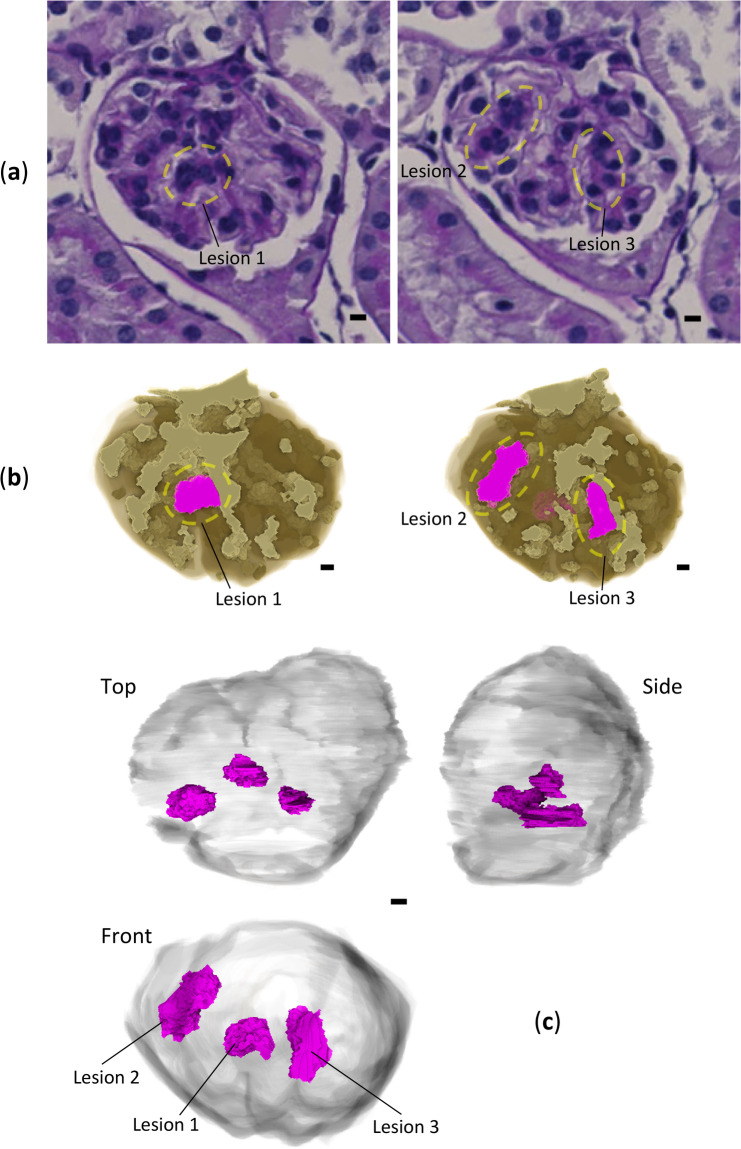
3D analysis of glomerular hypercellularity lesions. Scale bars: 5 μm. Measured volume for each lesion is given in Table 2. (**a**) Lesions on LM images. Three lesions are circled with yellow broken lines. (**b**) Corresponding volume-rendered XRM images of the glomerulus. The models are cut by the same corresponding planes as those in (**a**). Perspective and colouring are the same as those of the front and cut models in Fig. 4a, respectively. (**c**) Volume rendering of lesions in three orthogonal perspectives. The perspective of the front model is the same as that in (**a**, **b**). Surrounding translucent objects represent Bowman’s space. Figures (**a**) and the others were produced using ImageJ and Drishti, respectively.

Previous studies have reported the glomerular volume to be approximately 10^5^ μm^3^^14^, and our current findings are consistent with these reports. The overall volumetric comparison of high-density regions between disease-model and normal mice did not show a clear difference in the ratio versus whole glomerular volume (Table 2). This indicates that a local 3D evaluation is required to analyze glomerular hypercellularity using XRM. To identify the glomerular hypercellularity lesions in XRM images, confirmation using corresponding LM images of the same measurement points of the same sample was essential. As a result, three lesions with different 3D morphology were identified (Fig. 6): lesion 1 referred to as “simple type,” a simple protrusion from a branch of normal mesangial region; lesion 2 referred to as “fusion type,” a fusion of two or more protrusions from a branch of normal mesangial region; lesion 3 referred to as “cramp type,” a cramp-like protrusion that bridges different branches of normal mesangial region. These hypercellularity lesions were further evaluated quantitatively. The number of cells in the lesion was confirmed by LM images with clearly stained cell nuclei; lesions 1, 2 and 3 contained 5, 8 and 5 cells, respectively. Aggregation of four or more mesangial cells is used as a diagnostic criterion for kidney diseases^15^. From a volumetric analysis using XRM images, the volumes of lesions 1, 2 and 3 were quantified as 486, 825 and 497 μm^3^, respectively (Table 2). Therefore, a mesangial cell in the hypercellularity lesions occupies a volume of about 100 μm^3^ in the case of mouse kidney. To our knowledge, no studies have specifically examined the volume of individual mesangial cells. The measured volume of a single mesangial cell, approximately 0.01–0.1% of the total glomerular volume, aligns well with our general impression based on pathological experience. However, it should be noted that the lesions in this work comprise mesangial cells and mesangial matrices, and the increase in volume could be due to an increase in mesangial matrix rather than an increase in mesangial cell number.

## Discussion

Although a kidney biopsy represents only a portion of the entire kidney, a 3D evaluation covering the entire biopsy specimen could be useful for clinical diagnosis. Unfortunately, there is no single 3D observation modality that can evaluate pathological changes in entire biopsies at cellular-level spatial resolution to date. Thus, various correlative microscopies that combine multiple observation modalities to complement each other have been sought. The nondestructive nature of XRM makes it suitable for correlative microscopy. For instance, a combination of FIB-SEM and XRM visualized the 3D structure of a fly neuron at subcellular-level resolution with a wide field of view covering the whole brain^16^. However, this EM-XRM combination is too laborious to be applied to clinical diagnosis. There is another combination of LM and XRM, referred to as correlative visible-light and X-ray microscopy (CLXM). For instance, CLXM enabled the 3D evaluation of subcellular structures by a combination of visible-fluorescence and soft X-ray microscopies^17,18^. However, this approach is also laborious and is not applicable to the evaluation of a whole biopsy, because it uses synchrotron-based soft X-rays to observe a thin ice-embedded bio-specimen in a vacuum environment. In this work, we introduced a new form of CLXM referred to as “laboratory-based cellular-level CLXM” that enables practical 3D evaluation of a whole mouse kidney biopsy at cellular-level spatial resolution using a laboratory-based XRM. Importantly, this approach is highly adaptable to conventional histological procedures. This is because formalin-fixed paraffin-embedded (FFPE) samples in histology are commonly used for XRM observations, where paraffin acts as a negative contrast agent^10^. Furthermore, LM and laboratory-based XRM in this approach have a high complementarity in terms of tissue identification, sample distortion, resolution anisotropy and image contrast. For that reason, the laboratory-based cellular-level CLXM was used in this study to evaluate a kidney biopsy from a 5/6 nephrectomy model mouse, which revealed that combined use of both visible-light and X-ray microscopies proved effective for 3D quantitative assessment of glomerular hypercellularity.

Present results suggest a possibility for laboratory-based cellular-level CLXM in the quantitative clinical diagnosis of kidney diseases. For instance, since aggregation of four or more mesangial cells is used as a diagnostic criterion for kidney diseases^15^, a measured volume of high-density glomerular aggregation of 400 μm^3^ or more in XRM images may indicate a pathological change of hypercellularity if the volumetric proportion between mesangial cell and mesangial matrix is unchanged by pathogenesis. This possibility should be examined further in the future work using biopsy samples from human kidney. Currently, the identification of potential lesions in XRM images requires verification in corresponding LM images. In the future, if XRM can be used for preliminary observation of kidney biopsy specimens to identify lesions, followed by detailed local characterization using other examination methods, the utility of laboratory-based XRM in clinical kidney diagnosis is expected to increase. After accumulating a substantial number of clinical cases, it may be possible in the future to replace the LM observation in the diagnosis of kidney diseases by the XRM observation. Importantly, X-ray opacity measured by XRM generally reflects the electron density distribution within the sample, demonstrating the high versatility of this observation modality. Therefore, laboratory-based cellular-level CLXM observation of standard FFPE samples has potential to be applied to various other renal diseases and pathological lesions, as well as diseases of other organs. For instance, since the 5/6 nephrectomy model is typically recognized as a model of focal segmental glomerulosclerosis (FSGS)^19^, it would be interesting to examine segmental sclerotic lesions in the future. Our high-resolution 3D analysis enables precise measurement of mesangial areas and cell counts, representing a major advantage. At present, however, the analysis process is highly time-consuming due to the involvement of numerous manual procedures, which limits the feasibility of performing detailed measurements and comparative studies across multiple glomeruli. We are currently working on developing analytical methods that can efficiently measure and compare the volumes of a larger number of cells in a shorter acquisition time. Although still under development, we believe this approach holds promise as a valuable pathological indicator.

From a technical standpoint, reduction of acquisition time may be important for the widespread application of XRM for disease diagnosis in the future. For instance, much shorter exposure time may be allowed depending upon the size/contrast of lesions to be observed. Although cellular-level observation using Cu-target X-rays presented here has a limitation of sample size no more than 2 mm ^11^, larger samples may be observed at tissue-level resolutions using higher-energy X-rays^2,3^. In this study, the improvement of XRM imaging through drift correction and phase retrieval was essential for the CLXM observation (Fig. 1e,f). The drift correction is currently performed using diamond microparticles. However, since a considerable technical skill is required for attaching the microparticles to the sample, more user-friendly protocols need to be developed. On the other hand, precise orientational alignment of XRM images with corresponding LM images was also important for the CLXM observation, which was achieved using cell nuclei as positional markers. This image alignment procedure may be implemented in future XRM software. Although segmentation of XRM images was performed manually in this study, automated segmentation based on a process such as the active contour method^20^ or deep learning^21^ would be available in the future. For instance, automatic segmentation of maize embryo at very low CNR around 1.5 was reported^22^. The difficulty of tissue identification in XRM images might be improved by appropriate heavy-atom staining. For instance, osmium tetroxide staining to enable a segmentation of single nephron^23,24^, genetically targeted osmium tetroxide staining of Drosophila neurons^16^, and a tissue-specific heavy-atom staining to enhance visualization of cytoplasm^25^ were reported. This heavy atom staining technique, like the staining technique established in LM, might be a key technology of XRM in the future.

## Methods

### Mouse kidney material

Experiments were performed on 6- to 8-week-old male C57BL/6 mice that were housed under controlled environmental conditions and maintained with standard food and water. The mice weighed between 20 and 25 g. The 5/6 nephrectomy was performed in a procedure like the one described previously^26^. Briefly, the anesthetized mice were placed on a heating pad to maintain a constant body temperature (37 °C). Left flank incisions were made, and two-thirds of the mass of the left kidney was ablated. Seven days after left nephrectomy, the right kidney was removed. After the renal nephrectomy, the flank incisions were closed in two layers with silk sutures. The kidney samples from 5/6 nephrectomy mice were used 8 weeks after right nephrectomy. Kidney biopsy was performed on the remnant kidney. Anesthesia was induced using a triple anesthesia mixture consisting of Domitor (7.5 μg), Midazolam (40 μg) and Vetorphale (50 μg) in 100 μl of normal saline. The solution was administered intraperitoneally at a dosage of 0.1 ml per 10 g of body weight. This combination ensured sufficient sedation and analgesia for the procedures performed. Euthanasia was carried out by cervical dislocation under deep anesthesia induced by the triple anesthetic mixture. This method was selected to minimize pain and distress in accordance with institutional and international guidelines for the humane treatment of laboratory animals. All procedures used in the animal experiments complied with the standards set out in the guidelines for the care and use of laboratory animals of Kanazawa Medical University and were approved by the Research Center for Animal Life Science of Kanazawa Medical University (Approval number: 2020–25). All animals were also maintained and used under the ARRIVE guidelines.

Paraffin-embedded mouse kidney tissues prepared by standard procedures were used for analysis. Procedures for preparation of paraffin-embedded mouse kidney are as follows. Kidney samples from normal and 5/6 nephrectomy mice were taken using biopsy needles (ACECUT, TSK Laboratory, Tochigi, Japan). The kidney specimens were fixed in enough (more than 10 times the tissue volume) 10% buffered formalin fixatives for over 24 h at room temperature. For dehydration of tissues, a series of ethanol (70, 80, 90, 95 and 100% ethanol) and xylene were used. Finally, the tissues were embedded into paraffin blocks.

The analytical data presented here are based on the analysis of a single glomerulus from a representative mouse. Currently, the analysis process is extremely time-consuming, making it difficult to conduct detailed measurements and comparative studies involving multiple glomeruli. We are now exploring analytical methods that can automatically measure the volumes of multiple cells within a shorter time frame. Consequently, comparing cell volumes across different disease states and/or age groups remains a challenge for future research.

### Preparation of XRM sample

A paraffin block containing the unstained mouse kidney biopsy was first processed into a small (2.5 mm × 2.4 mm × 1.5 mm) paraffin piece containing half of the biopsy (Supplementary methods). Subsequent steps of the direct mounting were performed as reported^10^, except that microparticles from black chips of diamond-wire saw (putative diamond particles with a few microns in size) were attached to the specimen as positional markers (Fig. 1a). The attachment of markers was done by adding a trace amount of the black chips into the melted paraffin embedding the biopsy. A single attached microparticle that moves without leaving the field-of-view by X-ray scanning is sufficient for the marker purposes. Additional information for XRM sample preparation is available in supplementary information (Supplementary methods, Supplementary Fig. S1).

### XRM observation

X-ray projection data for XRM were collected using nano3DX (Rigaku Corporation, Tokyo, Japan) adopting quasi-parallel beam geometry, with a CCD detector (3296 × 2472 pixels) and a CMOS detector (6252 × 4176 pixels) for the disease-model mouse and the normal mouse, respectively (see Supplementary methods in detail). For the disease-model mouse, 3400 frames with a pixel size of 0.275 μm (L0270 (20x) lens, bin 1) and with the region-of-interest of 0.892 mm × 0.197 mm were taken for 57.7 h. For the normal mouse, 3600 frames with a pixel size of 0.188 μm (L0270 (20x) lens, bin 1) and with the region-of-interest of 0.963 mm × 0.407 mm were taken for 62.5 h. Rigid-body movement of the sample during data acquisition was evaluated in both vertical and horizontal directions using the positional marker. The positional shifts of the sample movement were corrected by the drift correction^27^, where the coordinates of projection images were translated to cancel the shifts (Supplementary methods). For the disease-model mouse, conventional flat-field correction, ring artifacts reduction^28^, vertical drift correction, propagation-based phase retrieval by Paganin method^29–32^ with parameter δ/β of 100, and conventional median filtering (radius 1 pixel) was applied in this order to the projection data. Similarly, for the normal mouse, median filtering (radius 1 pixel), flat-field correction, ring artifacts reduction, normalization of mean brightness by frame based on the histogram of a selected air region, vertical/horizontal drift correction, and phase retrieval with parameter δ/β of 100 was applied in this order to the projection data. CT reconstruction (16 bit) based on a conventional filtered-back-projection method (convolution back-projection) was performed with voxel sizes of 0.271 μm and 0.185 μm for the disease-model mouse and the normal mouse, respectively. The spatial resolution of a CT image was evaluated using the size of blurring at edges (SBE) from a line opacity profile across a paraffin-tissue boundary in a CT slice^10^. The contrast-to-noise ratio (CNR) between two regions in a CT slice was measured as reported previously^10^. The statistical significance on the difference between a pair of average CNR values was evaluated by a two-tailed Welch’s *t*-test under a null hypothesis of no difference; the equality of variances was examined in advance by the *F*-test at the significance level of 5% and found unequal; the accordance with a normal distribution was confirmed by the Kolmogorov–Smirnov test at the significance level of 5%. Although dimensional consistency of nano3DX system is not examined for this study, a magnification calibration factor of 1.01 for nano3DX was reported^33^ based on the intervals of the micro-line structures evaluated by a calibrated cross-sectional scanning electron microscopy.

### Visible-light microscope observation

After XRM observation, the biopsy sample was submitted to LM observation as follows. First, the biopsy sample was re-embedded in a standard paraffin block. For the pathological analysis, the paraffin block was cut at 4 μm and stained with periodic acid Schiff’s reagent (PAS). The PAS-stained tissues were digitally captured (Nano Zoomer C9600-03, Hamamatsu Photonics K.K, Hamamatsu, Japan) and used for further digital image analysis.

### Image analysis

Alignment of the XRM image (drift-corrected/phase-retrieved; 709 slices) with the LM image was performed manually using Drishti^34^ and ImageJ^35^ software. First, the file format of the LM image was converted to tiff using the “Ndpisplit” function of “NDPItools” plugin^36^ in ImageJ, and an approximate orientation of the XRM image was found using the “Cut” function in Drishti. Then, the orientation of the XRM image was aligned precisely with that of the LM image using cell nuclei as a positional marker (Supplementary methods) to produce the final XRM image for the XRM-LM comparison. Segmentation of a glomerulus was performed manually using ImageJ as reported^10^. Volume rendering was performed in Drishti. The volumetric analysis of glomerulus using ImageJ was performed as reported previously^10^, except that the binary region selected was smoothened by the “Open” and “Close” functions (once each in this order) in the case of the disease-model mouse.

## Supplementary Information

Below is the link to the electronic supplementary material.

Supplementary Material 1

Supplementary Material 2

Supplementary Material 3

Supplementary Material 4

Supplementary Material 5

Supplementary Material 6

Supplementary Material 7

Supplementary Material 8

Supplementary Material 9

Supplementary Material 10

Supplementary Material 11

Supplementary Material 12

Supplementary Material 13

Supplementary Material 14

Supplementary Material 15

Supplementary Material 16

## Data Availability

All data generated or analyzed in this study were included in the main text and in the supplementary information for this article.
