# Supplementary material for: Laboratory-based cellular-level correlative visible-light and X-ray microscopy for 3D evaluation of mouse kidney biopsy
Source: Sci Rep. 2026 Apr 2;16:15634. doi: 10.1038/s41598-026-44720-0 (PMC13187297; doi:10.1038/s41598-026-44720-0)
Supplement: Supplementary file 1 — Supplementary Material 1 [file 41598_2026_44720_MOESM1_ESM.pdf]

Supplementary information cover for “Laboratory-based cellular-level correlative visible-light and X-ray microscopy for 3D evaluation of mouse kidney biopsy”

Naoki Kunishima[1*], Raita Hirose[1], Yoshihiro Takeda[1], Koichiro Ito[2], Kengo Furuichi[3*] & Kazuhiko Omote[1]

[1]X-ray Research Laboratory and [2]New Market Development Office, Rigaku Corporation, 3-9-12 Matsubara-cho, Akishima, Tokyo 196-8666, Japan.

[3]Kanazawa Medical University, School of Medicine, Department of Nephrology, 1-1 Daigaku, Uchinada, Kahoku, Ishikawa 920-0293, Japan.

[*]email: kunisima@rigaku.co.jp; furuichi@kanazawa-med.ac.jp

Contents:

Supplementary information cover. Contents list of supplementary information. (41598_2026_44720_MOESM1_ESM.pdf; this file)

Video S1. Original CT image around glomerulus from disease-model mouse. (41598_2026_44720_MOESM2_ESM.avi; produced using ImageJ)

Video S2. Segmented CT image of glomerulus with Bowman’s capsule from disease-model mouse. (41598_2026_44720_MOESM3_ESM.avi; produced using ImageJ)

Video S3. Segmented CT image of glomerulus without Bowman’s capsule from disease-model mouse. (41598_2026_44720_MOESM4_ESM.avi; produced using ImageJ)

Video S4. High-density regions of CT image in glomerulus without Bowman's capsule from disease-model mouse. (41598_2026_44720_MOESM5_ESM.avi; produced using ImageJ)

Video S5. Segmented CT image of lesion 1 from disease-model mouse. (41598_2026_44720_MOESM6_ESM.avi; produced using ImageJ)

Video S6. Segmented CT image of lesion 2 from disease-model mouse. (41598_2026_44720_MOESM7_ESM.avi; produced using ImageJ)

Video S7. Segmented CT image of lesion 3 from disease-model mouse. (41598_2026_44720_MOESM8_ESM.avi; produced using ImageJ)

Video S8. Volume rendering model of segmented glomerulus with Bowman's capsule from disease-model mouse. (41598_2026_44720_MOESM9_ESM.avi; produced using Drishti)

Video S9. Original CT image around glomerulus from normal mouse. (41598_2026_44720_MOESM10_ESM.avi; produced using ImageJ)

Video S10. Segmented CT image of glomerulus with Bowman's capsule from normal mouse. (41598_2026_44720_MOESM11_ESM.avi; produced using ImageJ)

Video S11. Segmented CT image of glomerulus without Bowman's capsule from normal mouse. (41598_2026_44720_MOESM12_ESM.avi; produced using ImageJ)

Video S12. High-density regions of CT image in glomerulus without Bowman's capsule from normal mouse. (41598_2026_44720_MOESM13_ESM.avi; produced using ImageJ)

Video S13. Volume rendering model of segmented glomerulus with Bowman's capsule from normal mouse. (41598_2026_44720_MOESM14_ESM.avi; produced using Drishti)

Supplementary methods. Supplementary methods for this article. (41598_2026_44720_MOESM15_ESM.pdf)

Fig. S1. Additional information for XRM sample preparation. (41598_2026_44720_MOESM16_ESM.pdf)
