# Supplementary material for: Laboratory-based cellular-level correlative visible-light and X-ray microscopy for 3D evaluation of mouse kidney biopsy
Source: Sci Rep. 2026 Apr 2;16:15634. doi: 10.1038/s41598-026-44720-0 (PMC13187297; doi:10.1038/s41598-026-44720-0)
Supplement: Supplementary file 15 — Supplementary Material 15 [file 41598_2026_44720_MOESM15_ESM.pdf]

Supplementary methods

Preparation of XRM specimen

First, a paraffin block containing a kidney biopsy was manually trimmed to a smaller size of 5 mm × 4 mm using a disposable razor so that the embedded biopsy was positioned just below the top surface of the trimmed block (Supplementary Fig. S1a–c). Then the trimmed block was cut using a diamond wire saw CS-203 (Musashino Denshi Inc., Tokyo, Japan) with a custom-made sample holder allowing adjustment of the cutting angle (Tsukumo Engineering Inc., Saitama, Japan). The diameter of diamond wire used was 140 µm (120 µm core). After attachment on the wire saw, the top surface of the paraffin block was incised horizontally by 3 mm at a depth of 1.5 mm in parallel to the long axis of the cylindrical biopsy so that the biopsy was lifted from the remaining part of the paraffin block. Then, the wire saw was used further to make a 2 mm vertical incision in the block, cutting the embedded biopsy in half perpendicular to its long axis direction. Finally, a small (2.5 mm × 2.4 mm × 1.5 mm) paraffin piece containing half of the biopsy was manually cut from the rest of the block using a disposable knife (Supplementary Fig. S1d). Subsequent preparation steps to produce a specimen for XRM observation (Supplementary Fig. S1e) are described in the text.

XRM observation

The specimen set on the XRM apparatus was scanned by unfiltered X-rays from a rotating anode Cu-target (40 kV, 30 mA; spot size of 70 µm). The X-rays consisted mainly of characteristic X-rays at 8 keV and contained additional continuous X-rays. The source-to-sample distance was set at 260 mm. Projection data (0–180°) were collected in step scan mode with 60 s exposure per frame and with a sample-to-detector distance of 4 mm. The drift collection was performed as

follows. Vertical shifts were evaluated in consecutive regions of projection images including the marker. First, projections of pixels by minimum intensity in the horizontal direction were sorted versus frame number to produce a vertical marker trajectory (Fig. 1b). The vertical shifts were obtained by tracing the vertical marker trajectory. For the vertical drift correction, the coordinates of projection images were translated by adding compensation values to cancel the vertical shifts (Fig. 1d). After the vertical drift correction, horizontal shifts were evaluated in the same regions of projection images. First, projections of pixels by minimum intensity in the vertical direction were sorted versus frame number to produce a horizontal marker trajectory. After sine curve fitting, the horizontal marker trajectory was subtracted with the sine function to produce a residual horizontal marker trajectory (Fig. 1c). The horizontal shifts were obtained by tracing the residual horizontal marker trajectory. For the horizontal drift correction, the coordinates of projection images were translated by adding compensation values to cancel the horizontal shifts (Fig. 1d).

Image analysis

For the disease-model mouse, the XRM image was aligned with the LM image as follows. First, an approximate orientation of the original XRM image (3,291 × 3,291 × 709 pixels) was found using the "Cut" function in Drishti, and the XRM image was resliced so that the XRM image was approximately aligned with the LM image. In the resliced XRM image (2,439 × 555 × 1,791 pixels), a cell nucleus was selected as fiducial and ten other nuclei were selected as markers so that all eleven nuclei appeared on a LM section and the ten marker nuclei did not appear on the CT slice having the fiducial nucleus (fiducial slice). Then the resliced CT image was reoriented so that the marker nuclei appeared on the fiducial slice. The XRM image after the orientation refinement (2,445 × 1,008 × 1,986 pixels) was finally trimmed as 400 slices covering a glomerulus (slices

396–795/1,986; trimmed as a square 400 pixels on a side) for the XRM-LM comparison. All ten marker nuclei appeared on the fiducial slice after applying rotations of 2.6°, 2.6° and 39.1° around the x, y and z axes, respectively. For the ten marker nuclei after the orientation refinement, the average residuals in orientation angle between the plane of the fiducial slice and a line connecting the center position of a marker nucleus and the position of the fiducial nucleus on the fiducial slice was about 0.3°.

For the normal mouse, the alignment of the XRM image was performed similarly as follows. First, slices of the original XRM image (5,184 × 5,184 × 2,168 pixels) were trimmed as square 800 pixels on a side covering a glomerulus (slices 701–1,500/2,168) and were resliced so that the XRM image was approximately aligned with the LM image. Then, the orientation of the resliced image (800 × 800 × 800 pixels) was refined using a fiducial nucleus and ten marker nuclei, to produce the final XRM image (703 slices of a square 720 pixels on a side) for the XRM-LM comparison. After the orientation refinement, all ten marker nuclei appeared on the fiducial slice after applying rotations of 1.5°, -5.8° and -10.2°, around the x, y and z axes, respectively. For the ten marker nuclei, the average residual in orientation angle between the plane of the fiducial slice and a line connecting the center position of a marker nucleus and the position of the fiducial nucleus on the fiducial slice was about 0.7°.
