# Supplementary material for: Laboratory-based cellular-level correlative visible-light and X-ray microscopy for 3D evaluation of mouse kidney biopsy
Source: Sci Rep. 2026 Apr 2;16:15634. doi: 10.1038/s41598-026-44720-0 (PMC13187297; doi:10.1038/s41598-026-44720-0)
Supplement: Supplementary file 16 — Supplementary Material 16 [file 41598_2026_44720_MOESM16_ESM.pdf]

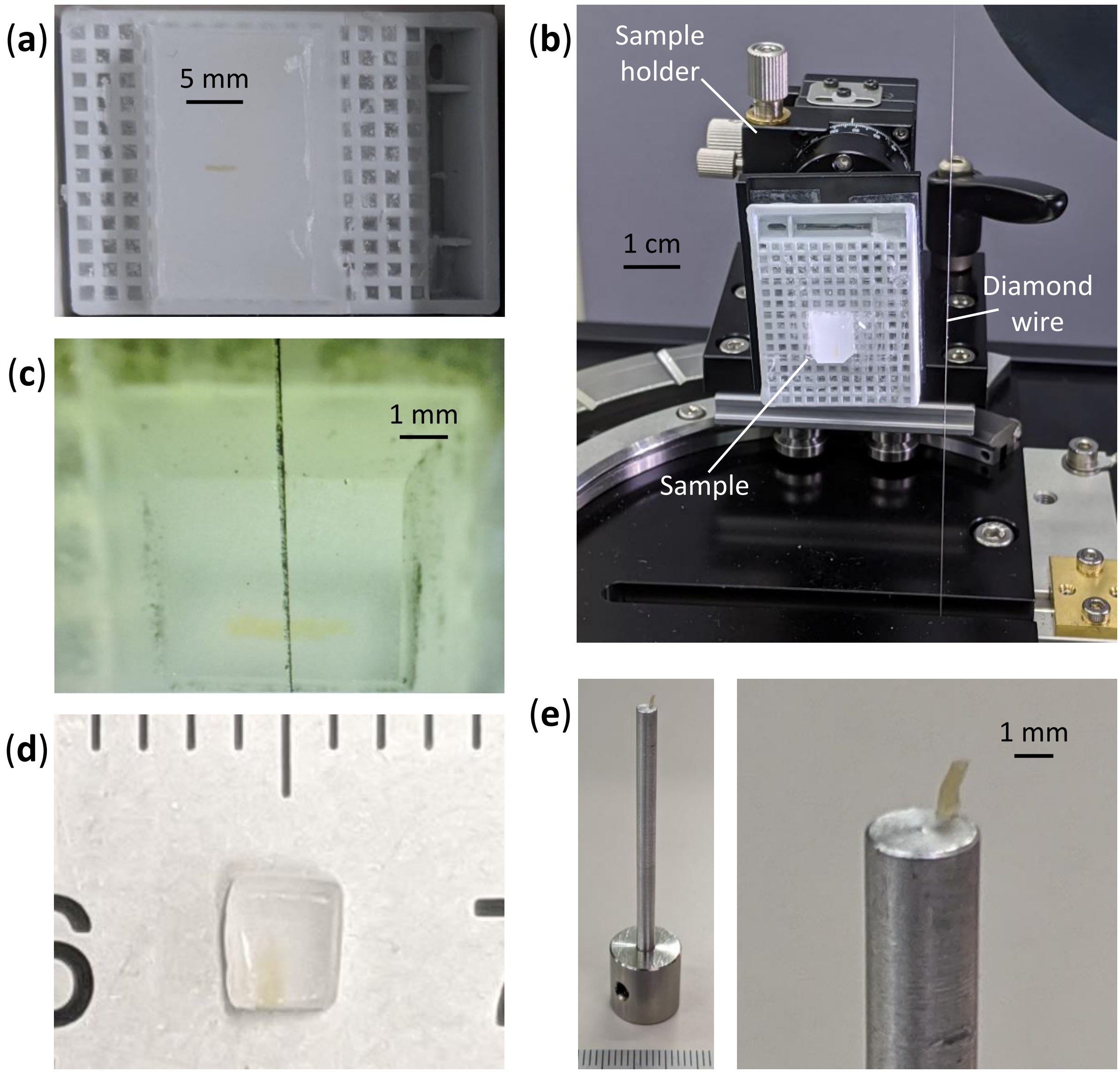

Supplementary Fig. S1. Additional information for XRM sample preparation. (**a**) Photograph of original paraffin block. Mouse kidney biopsy from a disease-model mouse is embedded around the top center of the paraffin block on an embedding cassette. (**b**) Configuration of sample cutting by diamond wire saw. Representative parts are labeled with names. Trimmed paraffin block is attached to the wire saw with a dedicated sample holder. (**c**) Close-up view of sample cutting. This photograph shows the status after the first horizontal cut and before the second vertical cut. The biopsy embedded is faintly visible at the bottom of the photograph as a yellow cylinder. Diamond wire is seen as a vertical linear object. (**d**) Piece of paraffin block after cutting. Half of the biopsy is faintly visible at the bottom of the block as a yellow cylinder with its long axis oriented vertically. (**e**) Photograph of direct-mounted mouse kidney biopsy: entire image (left) and magnified part around the specimen (right).
